# Associations of Coarse Grain Intake with Undiagnosed Hypertension among Chinese Adults: Results from the China Kadoorie Biobank

**DOI:** 10.3390/nu12123814

**Published:** 2020-12-13

**Authors:** Xin Liu, Hao Lai, Baibing Mi, Xin Qi, Wei Gan, Huaidong Du

**Affiliations:** 1 Department of Epidemiology and Biostatistics, School of Public Health, Global Health Institute, Xi’an Jiaotong University Health Science Center, 76 West Yanta Road, Xi’an 710061, China; lovemust1314@stu.xjtu.edu.cn (H.L.); xjtu.mi@xjtu.edu.cn (B.M.); xin.qi@xjtu.edu.cn (X.Q.); 2 Medical Research Council Population Health Research Unit, Nuffield Department of Population Health, University of Oxford, Oxford OX3 7LF, UK; wei.gan@ndph.ox.ac.uk (W.G.); huaidong.du@ndph.ox.ac.uk (H.D.); 3 Clinical Trial Service Unit and Epidemiological Studies Unit (CTSU), Nuffield Department of Population Health, University of Oxford, Oxford OX3 7LF, UK

**Keywords:** coarse grain, hypertension, blood pressure

## Abstract

Whole grain intake was associated with better blood pressure control, but evidence is lacking in non-Western populations with different grain intake patterns. We aimed to determine the associations between coarse grain intake, usually considered as the best proxy of whole grain intake for Chinese diets, with blood pressure and undiagnosed hypertension using baseline data from the China Kadoorie Biobank study. After excluding participants with clinically diagnosed hypertension or use of antihypertensive dugs, 435,907 participants were included in our analysis. A self-reported questionnaire was used to measure coarse grain intake frequency. Overall, 12.8% and 29.2% of the participants reported daily consumption and never consumption, respectively. With multivariable adjustments including BMI, outdoor temperature, and physical activity, higher frequency of coarse grain intake was associated with lower systolic and diastolic blood pressure in those older than 40 years, *p* trend < 0.05. Compared to never consumers, the odds ratio (95% CI) of hypertension was 0.78 (0.73–0.84), 0.84 (0.77–0.91), 0.91 (0.88–0.94), and 0.97 (0.95–0.99) for daily, 4–6 days/week, 1–3 days/week, and monthly groups, *P* trend < 0.001. Our cross-sectional study in a nationwide sample of Chinese adults suggests that higher coarse grain intake was associated with lower blood pressure and lower hypertension risk.

## 1. Introduction

Globally, elevated blood pressure was identified as a major modifiable risk factor for non-communicable diseases, such as stroke and ischemic heart disease [1]. A 25% reduction of elevated blood pressure by 2025 was set as a global target by the World Health Organization [2]. From the China Hypertension Survey 2012–2015, 23.2% of urban and rural adults were hypertensive, and 53.1% of them were unaware of their hypertensive status [3]. Most likely, there was no intentional medication or intervention on those people with undiagnosed hypertension. Many evidences were well recorded for the dietary patterns in the control of blood pressure, such as the Dietary Approaches to Stop Hypertension [4], and the Mediterranean Diet [5], both of which emphasize the intake of vegetables, fruit, and whole grains, with reduced intake of saturated fat and red meat. However, these dietary recommendations originated from Western populations, whose dietary pattern, cooking habits, and food supplies are different from those of Asians, including Chinese [6]. Therefore, the generalizability of those observed associations is still uncertain for the general population in China.

From recent studies, increased intake of whole grains was associated with reduced blood pressure [7,8,9,10]. Of note, the major resources of whole grains in Western diets are breads and breakfast cereals [11], while in Chinese diets, rice and wheat products are the major forms in grain consumption [12], often known as refined grain. Although brown rice was introduced as a whole grain option, its popularity is very limited given its high price, unique taste, and different cooking protocol [13]. In addition to refined grain, coarse grains (such as millet, corn, oats, sorghum, adlay, and buckwheat) are major alternative grain sources among the Chinese [14]. In the China National Nutrition and Health Surveillance, coarse grains were defined as grains or various beans (usually mung, red, or kidney beans) other than wheat or rice products [15]. Coarse grains share nutritional advantages with whole grains, such as richness in dietary fiber and B vitamins, which were related to improved endothelial functions and vascular health [7]. In a group of apparently healthy young adult Chinese with a mean age of 23 years, an inverse association between coarse grain intake and blood pressure has been reported previously [16]. However, whether this association exists in other age groups is still unknown. More importantly, the quantitative relevance of coarse grain intake for hypertension prevention in general population remains to be elucidated.

In the present study, we aim to determine the association between coarse grain intake frequency with blood pressure, and undiagnosed hypertension using cross-sectional data from a nationwide large-scale cohort in China.

## 2. Materials and Methods

### 2.1. Study Design and Participants

We utilized the baseline data of the China Kadoorie Biobank (CKB). The detailed study design has been described elsewhere [17,18]. In brief, the CKB study is a prospective cohort study among 0.5 million adult Chinese. Between 25 June 2004 and 15 July 2008, the baseline survey was conducted in ten areas in China (5 urban, 5 rural), which were selected from China’s nationally representative Disease Surveillance Points, to cover different socioeconomic levels, disease patterns, and associated risk factors. All registered residents at local study areas were invited to participate in the baseline survey if their age was between 35 to 74 years, and the average response rate was 30%. The final age range was 30 to 79 years after additional inclusion of about 2% of participants slightly outside the target age. The dataset used in the current study was release 15.0, including a total of 512,715 participants at baseline. In order to assess the association between coarse grain intake with undiagnosed hypertension, those who reported a diagnosis of hypertension by a clinician or use of anti-hypertensive drugs (n = 76,697) were excluded. In addition, participants with extreme blood pressure values (n = 109, systolic blood pressure (SBP) <80 or ≥250 mm Hg; diastolic blood pressure (DBP) <40 or ≥150 mm Hg) or having missing values (n = 2 for missing body mass index (BMI)) were also excluded, leaving 435,907 participants in the present study.

### 2.2. Data Collection

At the study clinics, information on sociodemographic status, lifestyle factors (such as smoking, alcohol drinking, and dietary habit), and medical history were collected via face-to-face interviews by trained health workers, using laptop-based questionnaires. With all participants wearing light clothes and no shoes, weight was measured using a bioelectrical impedance analysis instrument (TANITA-TBF-300GS; Tanita Corporation, Tokyo, Japan). Standing height was measured to the nearest 0.1 cm using a stadiometer and BMI was calculated as weight in kilograms divided by the square of height in meters (kg/m^2^). After at least five minutes’ rest, blood pressure was measured on the right upper arm at least twice in a seated position using a digital sphygmomanometer (model UA-779, A&D Medical, Tokyo, Japan) in a quiet and warm room. All devices were calibrated and measurements were done by trained staff according to a standard protocol to ensure measurement consistency. Normally, the cuff of the sphygmomanometer for adult size was used, while an alternative sphygmomanometer cuff size was used if arm circumference was outside the normal range, with a proper elasticity. The center of the cuff was approximately flush with the subject’s heart position. The average of the two measurements was used in the analysis. Because blood pressure is strongly associated with outdoor temperature, we also collected monthly mean outdoor temperature (MMOT) of each study area during baseline survey from the website of the China Meteorological Administration [19]. Each participant was assigned an MMOT according to the date when the baseline survey was done [20]. The study protocol was approved by the Oxford University Tropical Research Ethics Committee and the Chinese Center for Disease Control and Prevention Ethical Review Committee. All the participants provided written informed consent prior to participation.

### 2.3. Dietary Assessment

A simple food frequency questionnaire was used to collect consumption frequency of 12 major food groups, including the coarse grains [21]. It was expressed as “other staple food” (i.e., other than refined grain rice and wheat products) in some other published papers from the CKB study [22]; however, in the original Chinese questionnaire, it was expressed as “Za Liang”, with the same definition of coarse grains as the China National Nutrition and Health Surveillance [15]. The participants were required to answer the frequencies of food group consumption during the previous 12 months, by choosing one of the five categories (daily, 4 to 6 days per week, 1 to 3 days per week, monthly, or never or rarely (the reference category)). A validation study was conducted among a subset of the participants who complete the dietary questionnaire twice with an interval of less than 1.5 years, and results showed satisfying agreement between the questionnaire measurement and the average of 24 h recalls [23].

### 2.4. Definition of Outcomes

Undiagnosed hypertension was defined as having a measured SBP ≥ 140 mmHg or DBP ≥ 90 mmHg.

### 2.5. Statistical Analysis

Mean (SD) or number (percentage) of population characteristics were presented by coarse grain intake level. General linear model was used in comparing between-group differences for continuous variables, and chi-square test was used for categorical variables. Adjusted means of SBP and DBP were generated using general linear model by adjusting for age, sex, study area, education level (no formal education, primary school, middle/high school, or college/university), smoking (never, occasional, ex-regular, or regular), alcohol drinking (never, occasional, ex-regular, or regular), physical activity level (metabolic equivalent of task hours (MET-hr)/day), BMI, and MMOT where appropriate. Odds ratios (OR) of undiagnosed hypertension by coarse grain intake frequency were evaluated using logistic regression. Age, sex, and study area were included as covariates in model 1; education level, smoking, alcohol drinking, physical activity, BMI, and MMOT were additionally adjusted in model 2. SAS 9.3 (SAS Institute, Cary, NC, USA) was used in data analyses, and R Software (version 3.6.2; https://www.r-project.org) was used in plotting the figures. Two-sided *P* < 0.05 was considered as statistically significant.

## 3. Results

Among all our participants, 127,277 (29.2%) reported never or rarely consume coarse grains, while 55,997 (12.8%) reported daily consumption (Table 1). Daily consumers tended to be older and physically active, and to have lower frequencies in alcohol drinking and smoking, and lower SBP and DBP; while those choosing regular (4–6 days/week) intake but not daily, were more likely to be women or to live in urban areas.

**Figure 1:**
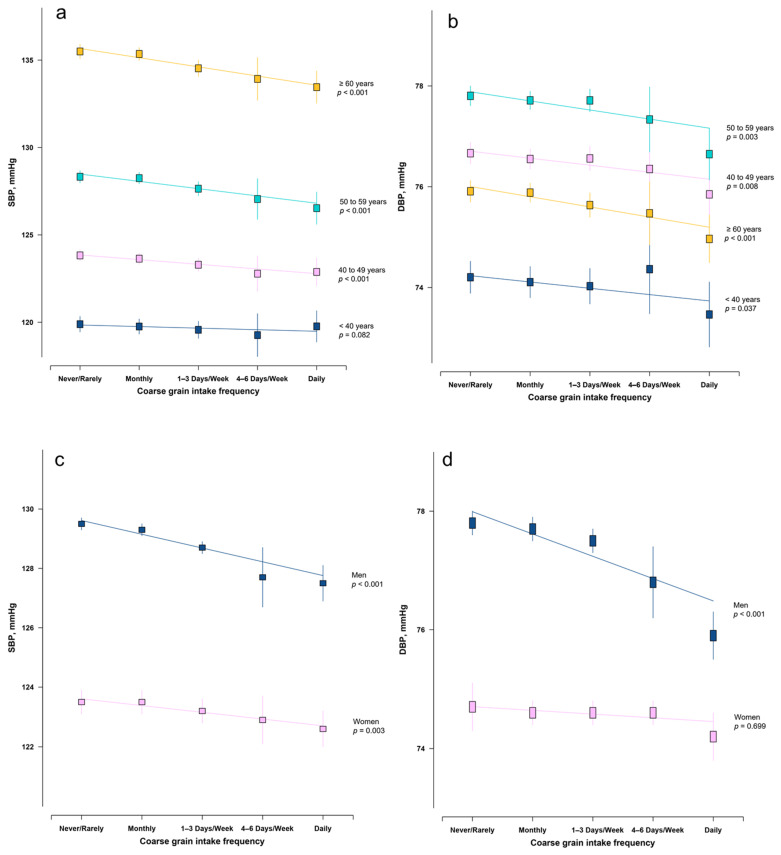
Adjusted systolic blood pressure (SBP) and diastolic blood pressure (DBP) by coarse grain intake frequency, age (**a**,**b**) and sex (**c**,**d**). Values were means adjusted for age, sex, study area, education level, smoking, alcohol drinking, physical activity level, body mass index (BMI), and local outdoor temperature.

The adjusted means of SBP and DBP by coarse grain intake level and age group are presented in the Figure 1. After multiple adjustment for potential confounders including physical activity, BMI, and MMOT, higher frequency of coarse grain intake was associated with lower SBP and DBP across all the age groups, with the strength of association being stronger among older participants (Figure 1a,b). In stratified analyses by sex, the association was stronger in men than in women (Figure 1c,d). By further checking the association by age and sex, we only observed significant coarse grain–blood pressure association among men aged 40 years or older but not in other groups (Supplemental Figure S1).

**Figure 2:**
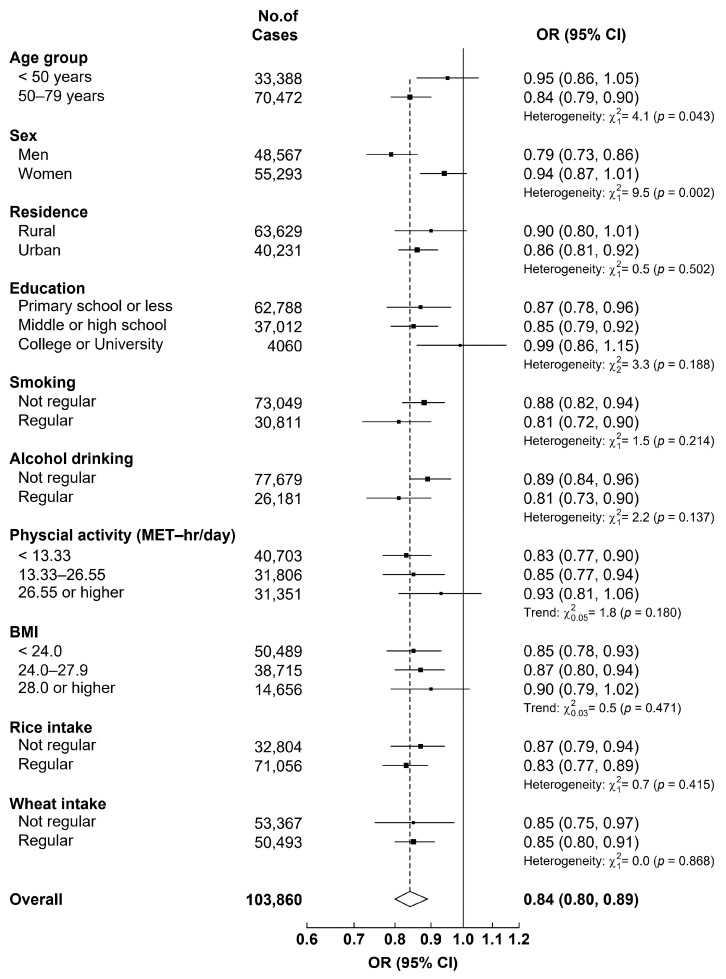
Adjusted odds ratios (ORs) of undiagnosed hypertension in those who have regular coarse grain intake (at least 4 days/week) vs. those who did not have by selected characteristics.

Coarse grain intake frequency was also inversely associated with the risk of undiagnosed hypertension (Table 2). After adjusting for age, sex, and study area, the OR (95% CI) of undiagnosed hypertension was 0.77 (0.72–0.82) for daily consumption, 0.86 (0.79–0.93) for 4–6 days/week consumption, 0.92 (0.89–0.94) for 1–3 days/week consumption, 0.97 (0.96–0.99) for monthly consumption group, as compared with never/rarely (null) consumption group, *p* for trend <0.001. The association was not substantially changed by further adjusting for education, smoking, alcohol drinking, physical activity level, BMI, and MMOT (OR [95% CI] comparing daily vs. null consumption groups, 0.78 [0.73–0.84], *P* for trend <0.001). The associations were similar in different age groups, but tended to be stronger among men and those living in urban areas, with ORs (95% CI) for daily consumption of 0.76 (0.70–0.823) and 0.74 (0.66–0.81), respectively, (*p* for interaction <0.05 for both, Table 2). However, when the participants were dichotomized into regular intake (at least 4 days/week) and non-regular intake ((less than 4 days/week)), we detected consistent inverse association between coarse grain intake category and undiagnosed hypertension by age, urban rural residence, smoking, physical activity, BMI level, and dietary intake frequency of rice and wheat products, while a sustained stronger association in men and in older people (Figure 2). We also examined this association by study area (Supplemental Figure S2) and similar direct associations were observed in 7 of the 10 areas. Moreover, we evaluated the association between coarse grain intake with blood pressure among those already diagnosed with hypertension within 3 years before baseline, and similar inverse association was observed (Supplemental Figure S3).

## 4. Discussion

In this nationwide cross-sectional study in Chinese people without clinically diagnosed hypertension, an independent inverse association was observed of coarse grain intake frequency with blood pressure and risk of hypertension. This association was stronger among men or older adults.

People diagnosed with hypertension, especially those without a proper control of blood pressure, are at higher risks of cardiovascular mortality [24], whereas usual SBP within a wide range (~120 to 180 mmHg) was continuously associated with elevated incidences of cardiovascular disease, and cardiovascular mortality, regardless of a diagnosis of hypertension, or use of anti-hypertensive drugs [2,25]. Protective factors of blood pressure need to be identified to minimize the future cardiovascular deteriorations attributable to elevated blood pressure among people without a clinical diagnosis of hypertension, given the relatively low diagnosis rate of hypertension [26], especially in developing countries such as China [24].

Several observational and interventional studies in Western populations showed that increased whole grain consumption was associated with reduced blood pressure or lower risk of hypertension [7,8,9,10]. In China, rice and wheat products are the major forms for grain intake, yet the use of brown rice and whole wheat or their products are of limited extent, partly owing to their availability, affordability, and unique mouthfeel [12,13]. By contrast, coarse grains, defined as grains or various beans (usually mung, red, or kidney beans) other than wheat or rice products, are very common in various food markets in China, but only 9.2%~14.6% of Chinese residents met the national recommendation of coarse grain intake level (50~100 g/day) according to the 2010–2012 China National Nutrition and Health Surveillance [15]. Given the shared nutrition advantages of whole grain and coarse grain, it would be of particular public health significance if beneficial effects of coarse grain intake on blood pressure control could be evidenced among the Chinese population.

In agreement with our pilot data in young adult Chinese (aged from 18 to 35 years) [16], the present study with a large nationwide population suggest that higher frequency of coarse grain intake was associated with lower blood pressure, across all groups (30 to 79 years). Furthermore, in our study participants without prior diagnosed hypertension, daily consumption of coarse grains was associated with a 22% lower risk of being diagnosed with hypertension, compared to those with never or rarely consumption. Similarly, 16% lower hazards ratio (0.84 [95% CI, 0.76–0.93], quartile 4 versus quartile 1) of incident hypertension comparing highest vs. lowest whole grain intake was reported in a study of 80,426 French adults participating in the NutriNetSanté cohort study [7]. In addition, a single-arm trial among 45 patients with mild hypertension also showed that a 12 week substitution of staple food by 50 g/day foxtail millet (a major coarse grain in China) may reduce blood pressure by 4 to 5 mmHg [27]. Taken together, these observations may advance our knowledge of the potential preferable association of coarse grain consumption with blood pressure control among general population in broad age range and blood pressure range, which has not been reported before.

Intriguingly in stratified analysis by gender, the association between coarse grain intake with blood pressure and undiagnosed hypertension was stronger in men than in women. In the previous studies on the association between whole grain intake with blood pressure and hypertension, no gender-specific results were reported [7,8,9,10]. However, in the Nurses’ Health Study and the Health Professionals Follow-up Study, independent inverse associations between dietary fiber and potassium with blood pressure were only observed in men, rather than in women [28,29]. Coarse grains, such as millet, corn, and buckwheat are rich in dietary fiber and potassium. Although we were not able to check whether the stronger association of coarse grains with blood pressure in men was driven by dietary fiber or potassium, the renin–angiotensin–aldosterone system, a major physiological system for blood pressure control, was highly influenced by sex hormones and menopause status in women [30], and was shown to be downregulated in hypertensive mice fed with a high-fiber diet in favor of preventing the onset of hypertension [31]. Those observations may partly explain the heterogeneity by sex observed in our analysis.

The exact mechanisms underlying the association between coarse grain intake frequency and blood pressure are not clear to us, although a few intervention studies suggested that dietary fiber supplementation may effectively reduce blood pressure in patients with hypertension [32,33]. Meanwhile, experimental biology evidence suggest that a high-fiber diet may alter the gut microbiota toward a protective profile in the development of cardiovascular disease and may change the gene network of the renin–angiotensin–aldosterone system [16], which is fundamental in blood pressure homeostasis. With fasting blood samples of our prior pilot study, we conducted untargeted metabolomic and lipidomic profiling. Among the identified metabolites predicting blood pressure, sphingolipid ceramides, triacylglycerols, phosphatidylcholines, and phosphatidylethanolamine were the major component that mediated the associations between coarse grain intake with blood pressure [34]. Moreover, data from the Framingham Offspring Cohort suggested that increasing whole grain consumption was associated with circulating sphingolipid ceramides [35]; evidence from animal models suggested sphingolipid de novo biosynthesis was actively involved in cardiovascular homeostasis, mediating vasoconstriction and blood pressure [36,37]. Obviously, further interventional studies are needed to elucidate the effectiveness of increasing coarse grain intake in blood pressure control and the underlining metabolomic pathways.

Our study has several limitations. Firstly, with a cross-sectional design, temporal association could not be established; despite people with a known status of hypertension having been excluded from analyses, the main results were not substantially changed in a sensitivity analysis by further excluding those diagnosed with diabetes and cardiovascular disease, or those in the Henan area, where 99% of participants reported daily consumption (Supplemental Table S1); secondly, the intake amount of coarse grains at each occasion was not available in the current study, therefore we were not able to evaluate the associations between intake amount with blood pressure, neither could we adjust for total energy intake in the model. However, after adjusting for BMI and physical activity level, two of the three parameters in the energy balance equation, the associations were not substantially changed. Moreover, a potential collinearity between increased coarse grain intake with reduced salt intake may also have biased the observed associations, which was to be elucidated in future. The strengths of our study may include the following: large nationwide sample enables us to evaluate detailed association patterns by age group, which is particularly critical for blood pressure control given their strong linear association with blood pressure and cardiovascular diseases incidence and mortality [2]; reverse causation bias caused by lifestyle modification after diagnosis may be minimized by excluding clinically diagnosed hypertension cases; individual level data of MMOT was adjusted for, thus the potential confounding effects of temperature could be minimized. Whole grain was shown to be prospectively associated with lower risks of coronary heart disease and stroke compared to Western populations [11]. With similar nutrient advantages to whole gains, coarse grain intake may be also related to reductions in risks of other cardiovascular diseases closely related to elevated blood pressure, which require further prospective studies.

## 5. Conclusions

In summary, with data from more than 0.4 million adult Chinese free of diagnosed hypertension, inverse independent associations were identified of coarse grain intake frequency with blood pressure and risk of undiagnosed hypertension. Our results may provide insights into further interventional and mechanism studies addressing the beneficial effects of coarse grain consumption on vascular health, especially among Chinese populations without a diagnosis of hypertension, and in regions where coarse grains are well-available and popular. Furthermore, to promote public awareness of potential cardiometabolic benefits of coarse grain intake, new interventional approaches (e.g., social media, school education) deserve further verification.

## Figures and Tables

**Table 1:** Characteristics of the participants *.

Characteristic	Never or Rarely	Monthly	1–3 Days/Week	4–6 Days/Week	Daily	All Participants
*n*	127,277	194,325	54,693	3615	55,997	435,907
Age (years)	49.6 (12.2)	50.4 (12.6)	51.5 (11.3)	53.8 (10.4)	56.2 (30.6)	50.8 (10.4)
Female (%)	55.4	60.5	62.4	65.8	56.2	58.7
Urban residence (%)	37.8	46.9	75.9	83.1	7.0	43.0
Education level (%)						
No formal education	20.9	18.7	11.4	10.3	11.6	17.4
Primary school	36.5	32.5	17.9	15.2	33.2	31.8
Middle or high school	39.2	43.2	55.1	55.6	52.9	44.9
College or university	3.4	5.6	15.6	18.8	2.3	5.9
Household Income (%)						
<10,000 yuan/year	29.9	27.4	21.2	17.9	39.1	28.8
10,000–19,999 yuan/year	25.7	27.5	28.3	30.2	43.4	29.1
20,000–34,999 yuan/year	25.1	26.1	29.2	29.5	13.1	24.5
≥35,000 yuan/year	19.3	19.1	21.4	22.4	4.4	17.6
Smoking status (%)						
Never	56.3	63.5	67.3	69.6	60.2	61.5
Occasional	5.5	5.5	5.1	4.8	8.1	5.8
Ex-regular	5.6	4.8	5.9	6.4	5.7	5.3
Regular	32.7	26.2	21.6	19.2	26.1	27.4
Alcohol drinking (%)						
Never	47.9	50.6	41.4	40.0	18.6	44.4
Occasional	24.7	29.9	36.8	38.6	59.4	33.1
Ex-regular	2.0	1.5	1.0	0.8	0.4	1.4
Regular	25.5	18.0	20.9	20.7	21.6	21.1
Physical activity (MET-hr/day)	19.4 (17.3)	19.2 (19.0)	19.3 (14.6)	19.3 (12.2)	19.7 (36.3)	21.9 (13.9)
BMI (kg/m^2^)	23.1 (4.5)	23.2 (4.9)	23.2 (3.8)	23.3 (3.2)	23.1 (9.5)	23.4 (3.3)
SBP (mmHg)	130.0 (25.4)	129.9 (27.8)	129.3 (21.4)	128.8 (17.9)	128.2 (53.2)	127.5 (18.9)
DBP (mmHg)	76.1 (14.6)	76.0 (16.0)	76.0 (12.3)	75.8 (10.3)	74.9 (30.6)	76.4 (10.4)

* Means (SD) standardized for age, sex and study area when appropriate. Categorical variables were shown as number (%). BMI, body mass index; DBP, diastolic blood pressure; SBP, systolic blood pressure; MET, metabolic equivalent task. Values were all significantly different among groups (*p* < 0.001).

**Table 2:** Adjusted odds ratios of undiagnosed hypertension by coarse grain intake frequency and stratified results by age, sex, and residence.

	Odds Ratio (95% CI)					*p* for Trend
	Never or Rarely	Monthly	1–3 Days/Week	4–6 Days/Week	Daily	
All participants						
Model 1	1.00	0.97 (0.96–0.99)	0.92 (0.89–0.94)	0.86 (0.79–0.93)	0.77 (0.72–0.82)	<0.001
Model 2	1.00	0.97 (0.95–0.99)	0.91 (0.88–0.94)	0.84 (0.77–0.91)	0.78 (0.73–0.84)	<0.001
<50.1 years (Median)						
Model 1	1.00	0.95 (0.93–0.98)	0.91 (0.87–0.95)	0.82 (0.70–0.96)	0.85 (0.75–0.95)	<0.001
Model 2	1.00	0.96 (0.93–0.99)	0.93 (0.89–0.97)	0.83 (0.70–0.98)	0.95 (0.84–1.07)	0.001
≥50.1 years (Median)						
Model 1	1.00	0.99 (0.97–1.02)	0.94 (0.91–0.97)	0.90 (0.82–1.00)	0.77 (0.71–0.84)	<0.001
Model 2	1.00	0.98 (0.96–1.01)	0.92 (0.89–0.96)	0.88 (0.79–0.97)	0.76 (0.70–0.83)	<0.001
Men						
Model 1	1.00	0.97 (0.95–1.00)	0.92 (0.88–0.96)	0.81 (0.71–0.92)	0.71 (0.64–0.78)	<0.001
Model 2	1.00	0.97 (0.94–0.99)	0.90 (0.86–0.94)	0.79 (0.69–0.91)	0.74 (0.66–0.81)	<0.001
Women						
Model 1	1.00	1.00 (0.98–1.03)	0.99 (0.95–1.03)	1.00 (0.90–1.11)	0.92 (0.84–1.01)	0.267
Model 2	1.00	0.99 (0.97–1.02)	0.97 (0.93–1.00)	0.95 (0.85–1.07)	0.90 (0.81–0.99)	0.020
Rural						
Model 1	1.00	1.01 (0.99–1.04)	1.03 (0.98–1.08)	1.03 (0.85–1.24)	0.84 (0.74–0.95)	0.911
Model 2	1.00	0.98 (0.96–1.01)	0.95 (0.91–1.00)	0.95 (0.77–1.16)	0.86 (0.75–0.98)	0.008
Urban						
Model 1	1.00	0.95 (0.92–0.98)	0.88 (0.85–0.91)	0.83 (0.76–0.92)	0.75 (0.69–0.82)	<0.001
Model 2	1.00	0.97 (0.94–1.00)	0.91 (0.88–0.95)	0.86 (0.78–0.95)	0.79 (0.73–0.86)	<0.001

Model 1, adjusted for age, sex, and study area; model 2, additionally adjusted for education level (no formal education, primary school, middle/high school, or college/university), smoking (never, occasional, ex-regular, or regular), alcohol drinking (never, occasional, ex-regular, or regular), physical activity (MET-hr/day), BMI, and local outdoor temperature; All *P* for interactions were significant (*p* < 0.05) except for the interaction with age group (*p* = 0.054 with model 2).

## Supplementary Materials

The following are available online at https://www.mdpi.com/2072-6643/12/12/3814/s1, Supplemental Figure S1. Adjusted ORs of undiagnosed hypertension in those who have regular coarse grain intake (at least 4 days/week) vs. those who had no intake by study area. Supplemental Figure S2 Adjusted ORs of undiagnosed hypertension in those who have regular coarse grain intake (at least 4 Days/Weeks) vs. those who didn’t have by study area. Supplemental Figure S3 Adjusted SBP and DBP by coarse grain intake frequency among diagnosed hypertension patients within 3 years before baseline (n = 34163). Values were means adjusted for age, study area, education level, smoking, alcohol drinking, physical activity level, BMI, and local outdoor temperature. Supplemental Table S1. Adjusted odds ratios of undiagnosed hypertension by coarse grain intake frequency with additional exclusion criteria.

## References

[1] Olsen M.H., Angell S.Y., Asma S., Boutouyrie P., Burger D., Chirinos J.A., Damasceno A., Delles C., Gimenez-Roqueplo A.P., Hering D. (2016). A call to action and a lifecourse strategy to address the global burden of raised blood pressure on current and future generations: The Lancet Commission on hypertension. Lancet.

[2] Lacey B., Lewington S., Clarke R., Kong X.L., Chen Y.P., Guo Y., Yang L., Bennett D., Bragg F., Bian Z. (2018). Age-specific association between blood pressure and vascular and non-vascular chronic diseases in 0.5 million adults in China: A prospective cohort study. Lancet Glob. Health.

[3] Wang Z.W., Chen Z., Zhang L.F., Wang X., Hao G., Zhang Z.G., Shao L., Tian Y., Dong Y., Zheng C.Y. (2018). Status of Hypertension in China Results From the China Hypertension Survey, 2012–2015. Circulation.

[4] Saneei P., Salehi-Abargouei A., Esmaillzadeh A., Azadbakht L. (2014). Influence of Dietary Approaches to Stop Hypertension (DASH) diet on blood pressure: A systematic review and meta-analysis on randomized controlled trials. Nutr. Metab. Cardiovasc. Dis. NMCD.

[5] Nissensohn M., Roman-Vinas B., Sanchez-Villegas A., Piscopo S., Serra-Majem L. (2016). The Effect of the Mediterranean Diet on Hypertension: A Systematic Review and Meta-Analysis. J. Nutr. Educ. Behav..

[6] Shono C., Suzuki N., Kaiser H.M. (2000). Will China’s diet follow western diets?. Agribus. Int. J..

[7] Lelong H., Blacher J., Baudry J., Adriouch S., Galan P., Fezeu L., Hercberg S., Kesse-Guyot E. (2017). Individual and Combined Effects of Dietary Factors on Risk of Incident Hypertension: Prospective Analysis from the NutriNet-Sante Cohort. Hypertension.

[8] Vernay M., Aidara M., Salanave B., Deschamps V., Malon A., Oleko A., Mallion J.M., Hercberg S., Castetbon K. (2012). Diet and blood pressure in 18–74-year-old adults: The French Nutrition and Health Survey (ENNS, 2006–2007). J. Hypertens..

[9] Kirwan J.P., Malin S.K., Scelsi A.R., Kullman E.L., Navaneethan S.D., Pagadala M.R., Haus J.M., Filion J., Godin J.P., Kochhar S. (2016). A Whole-Grain Diet Reduces Cardiovascular Risk Factors in Overweight and Obese Adults: A Randomized Controlled Trial. J. Nutr..

[10] Ampatzoglou A., Atwal K.K., Maidens C.M., Williams C.L., Ross A.B., Thielecke F., Jonnalagadda S.S., Kennedy O.B., Yaqoob P. (2015). Increased whole grain consumption does not affect blood biochemistry, body composition, or gut microbiology in healthy, low-habitual whole grain consumers. J. Nutr..

[11] Aune D., Keum N., Giovannucci E., Fadnes L.T., Boffetta P., Greenwood D.C., Tonstad S., Vatten L.J., Riboli E., Norat T. (2016). Whole grain consumption and risk of cardiovascular disease, cancer, and all cause and cause specific mortality: Systematic review and dose-response meta-analysis of prospective studies. BMJ.

[12] Villegas R., Liu S., Gao Y.T., Yang G., Li H., Zheng W., Shu X.O. (2007). Prospective study of dietary carbohydrates, glycemic index, glycemic load, and incidence of type 2 diabetes mellitus in middle-aged Chinese women. Arch. Intern. Med..

[13] Zhang G., Pan A., Zong G., Yu Z., Wu H., Chen X., Tang L., Feng Y., Zhou H., Chen X. (2011). Substituting white rice with brown rice for 16 weeks does not substantially affect metabolic risk factors in middle-aged Chinese men and women with diabetes or a high risk for diabetes. J. Nutr..

[14] He Y., Zhao L., Yu D., Xia J., Yang X., Yang Y. (2016). The coarse food intake and its impact on dietary nutrients in Chinese adults. Acta Nutr. Sin..

[15] He Y., Zhao L., Yu D., Hu J., Yang Y., Yang X. (2016). The status of coarse food intake among Chinese adults. Acta Nutr. Sin..

[16] Liu X., Liao X., Gan W., Ding X., Gao B., Wang H., Zhao X., Liu Y., Feng L., Abdulkadil W. (2019). Inverse Relationship between Coarse Food Grain Intake and Blood Pressure among Young Chinese Adults. Am. J. Hypertens..

[17] Chen Z., Lee L., Chen J., Collins R., Wu F., Guo Y., Linksted P., Peto R. (2005). Cohort profile: The Kadoorie Study of Chronic Disease in China (KSCDC). Int. J. Epidemiol..

[18] Chen Z., Chen J., Collins R., Guo Y., Peto R., Wu F., Li L., China Kadoorie Biobank Collaborative Group (2011). China Kadoorie Biobank of 0.5 million people: Survey methods, baseline characteristics and long-term follow-up. Int. J. Epidemiol..

[19] Yu H.-T., Fu X.-Y., Xu B., Zuo L.-l., Ma H.-B., Wang S.-R. (2018). Untargeted metabolomics approach (UPLC-Q-TOF-MS) explores the biomarkers of serum and urine in overweight/obese young men. Asia Pac. J. Clin. Nutr..

[20] Lewington S., Li L., Sherliker P., Guo Y., Millwood I., Bian Z., Whitlock G., Yang L., Collins R., Chen J. (2012). Seasonal variation in blood pressure and its relationship with outdoor temperature in 10 diverse regions of China: The China Kadoorie Biobank. J. Hypertens..

[21] Li L.M., Lv J., Guo Y., Collins R., Chen J.S., Peto R., Wu F., Chen Z.M., China Kadoorie Biobank Collaborative Group (2012). The China Kadoorie Biobank: Related methodology and baseline characteristics of the participants. Zhonghua Liu Xing Bing Xue Za Zhi.

[22] Du H., Li L., Bennett D., Guo Y., Key T.J., Bian Z., Sherliker P., Gao H., Chen Y., Yang L. (2016). Fresh Fruit Consumption and Major Cardiovascular Disease in China. N. Engl. J. Med..

[23] Lv J., Qi L., Yu C., Yang L., Guo Y., Chen Y., Bian Z., Sun D., Du J., Ge P. (2015). Consumption of spicy foods and total and cause specific mortality: Population based cohort study. BMJ.

[24] Lewington S., Lacey B., Clarke R., Guo Y., Kong X.L., Yang L., Chen Y., Bian Z., Chen J., Meng J. (2016). The Burden of Hypertension and Associated Risk for Cardiovascular Mortality in China. JAMA Intern. Med..

[25] Sundstrom J., Neovius M., Tynelius P., Rasmussen F. (2011). Association of blood pressure in late adolescence with subsequent mortality: Cohort study of Swedish male conscripts. BMJ.

[26] Wall H.K., Hannan J.A., Wright J.S. (2014). Patients with undiagnosed hypertension: Hiding in plain sight. JAMA.

[27] Hou D., Chen J., Ren X., Wang C., Diao X., Hu X., Zhang Y., Shen Q. (2018). A whole foxtail millet diet reduces blood pressure in subjects with mild hypertension. J. Cereal Sci..

[28] Ascherio A., Rimm E.B., Giovannucci E.L., Colditz G.A., Rosner B., Willett W.C., Sacks F., Stampfer M.J. (1992). A prospective study of nutritional factors and hypertension among US men. Circulation.

[29] Witteman J.C., Willett W.C., Stampfer M.J., Colditz G.A., Sacks F.M., Speizer F.E., Rosner B., Hennekens C.H. (1989). A prospective study of nutritional factors and hypertension among US women. Circulation.

[30] Komukai K., Mochizuki S., Yoshimura M. (2010). Gender and the renin-angiotensin-aldosterone system. Fundam. Clin. Pharmacol..

[31] Marques F.Z., Nelson E., Chu P.Y., Horlock D., Fiedler A., Ziemann M., Tan J.K., Kuruppu S., Rajapakse N.W., El-Osta A. (2017). High-Fiber Diet and Acetate Supplementation Change the Gut Microbiota and Prevent the Development of Hypertension and Heart Failure in Hypertensive Mice. Circulation.

[32] Keenan J.M., Pins J.J., Frazel C., Moran A., Turnquist L. (2002). Oat ingestion reduces systolic and diastolic blood pressure in patients with mild or borderline hypertension: A pilot trial. J. Fam. Pract..

[33] Aleixandre A., Miguel M. (2016). Dietary fiber and blood pressure control. Food Funct..

[34] Liu X., Shi L., Dai X., Chen H., Zhang C., Wang P., Wu Q., Zeng L., Yan H. (2020). Plasma metabolites mediate the association of coarse grain intake with blood pressure in hypertension-free adults. Nutr. Metab. Cardiovasc. Dis. NMCD.

[35] Walker M., Xanthakis V., Ma J., Quatromoni P.A., Moore L., Ramachandran V., Jacques P. (2019). A Mediterranean Style Diet Is Favorably Associated with Concentrations of Circulating Ceramides and Ceramide Ratios in the Framingham Offspring Cohort (P18-048-19). Curr. Dev. Nutr..

[36] Sasset L., Zhang Y., Dunn T.M., Di Lorenzo A. (2016). Sphingolipid De Novo Biosynthesis: A Rheostat of Cardiovascular Homeostasis. Trends Endocrinol. Metab. TEM.

[37] Fenger M., Linneberg A., Jorgensen T., Madsbad S., Sobye K., Eugen-Olsen J., Jeppesen J. (2011). Genetics of the ceramide/sphingosine-1-phosphate rheostat in blood pressure regulation and hypertension. BMC Genet..

