# Supplementary material for: Associations of Coarse Grain Intake with Undiagnosed Hypertension among Chinese Adults: Results from the China Kadoorie Biobank
Source: Nutrients. 2020 Dec 13;12(12):3814. doi: 10.3390/nu12123814 (PMC7764616; doi:10.3390/nu12123814)
Supplement: Supplementary file 1 [file nutrients-12-03814-s001.pdf]

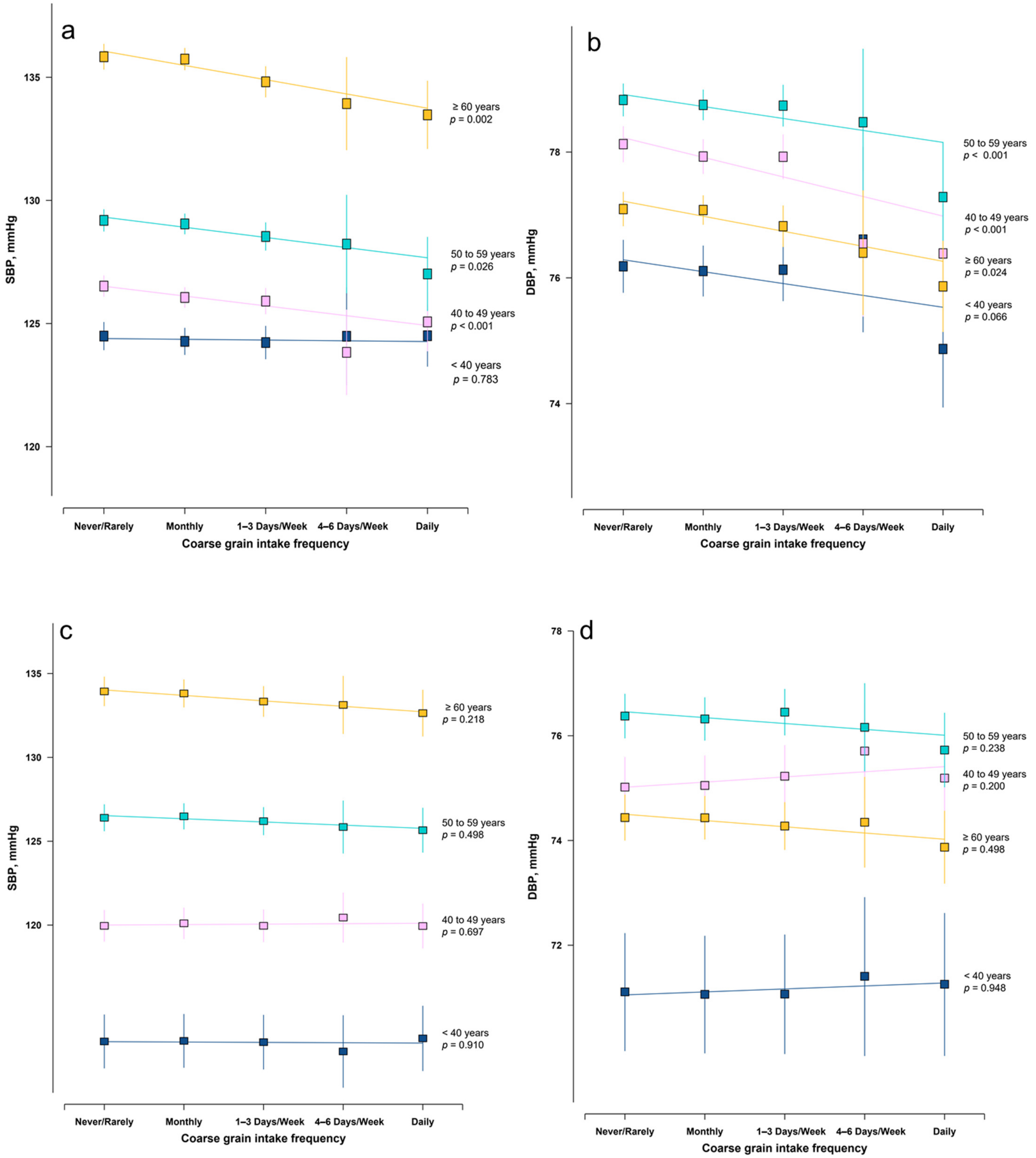

Supplemental Figure S1 Adjusted SBP and DBP by coarse grain intake frequency and age group in men (a, b) and women (c, d). Values were means adjusted for age, study area, education level, smoking, alcohol drinking, physical activity level, BMI, and local outdoor temperature.

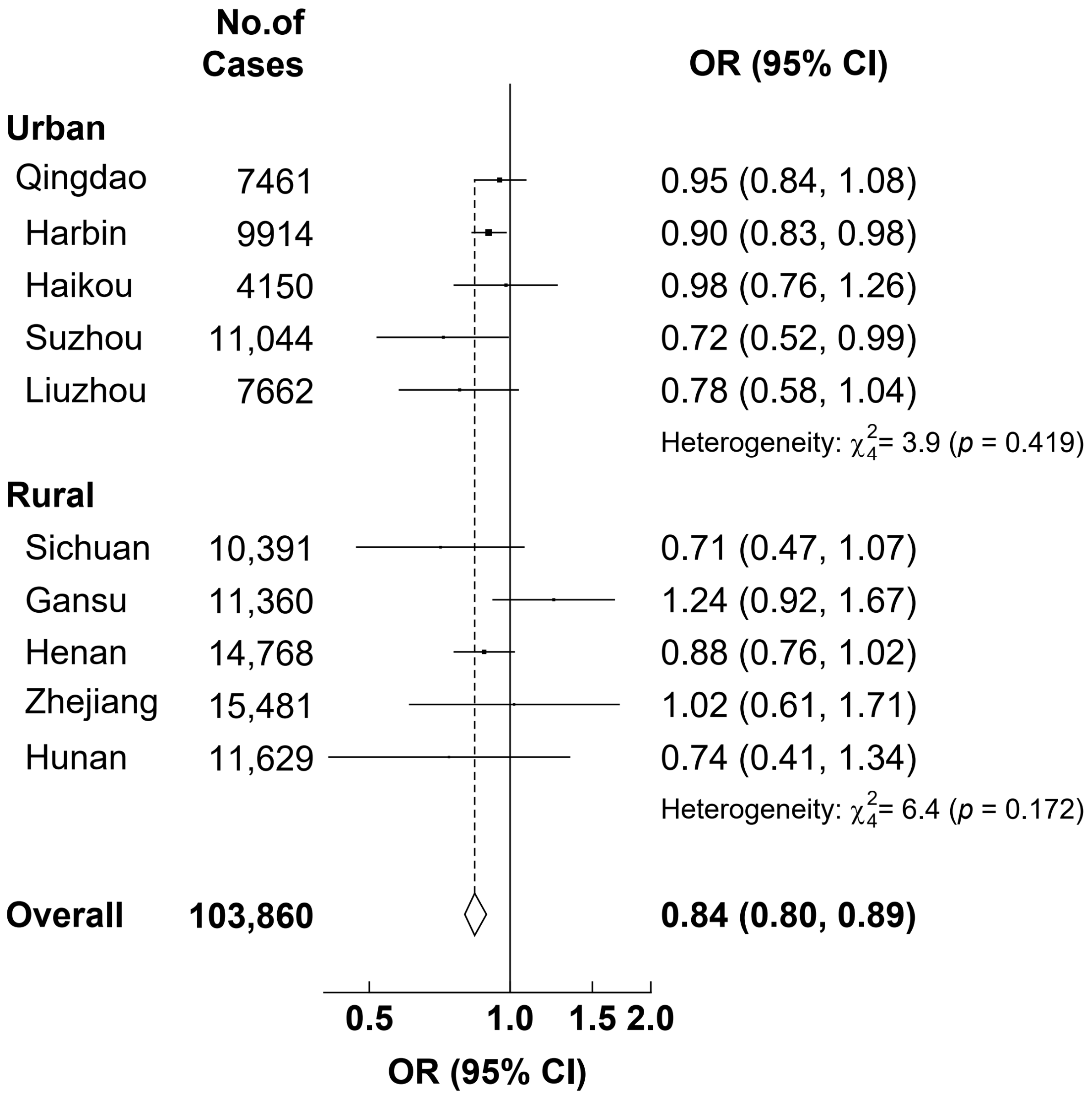

Supplemental Figure S2 Adjusted ORs of undiagnosed hypertension in those who have regular coarse grain intake (at least 4 Days/Wk) vs. those who didn't have by study area.

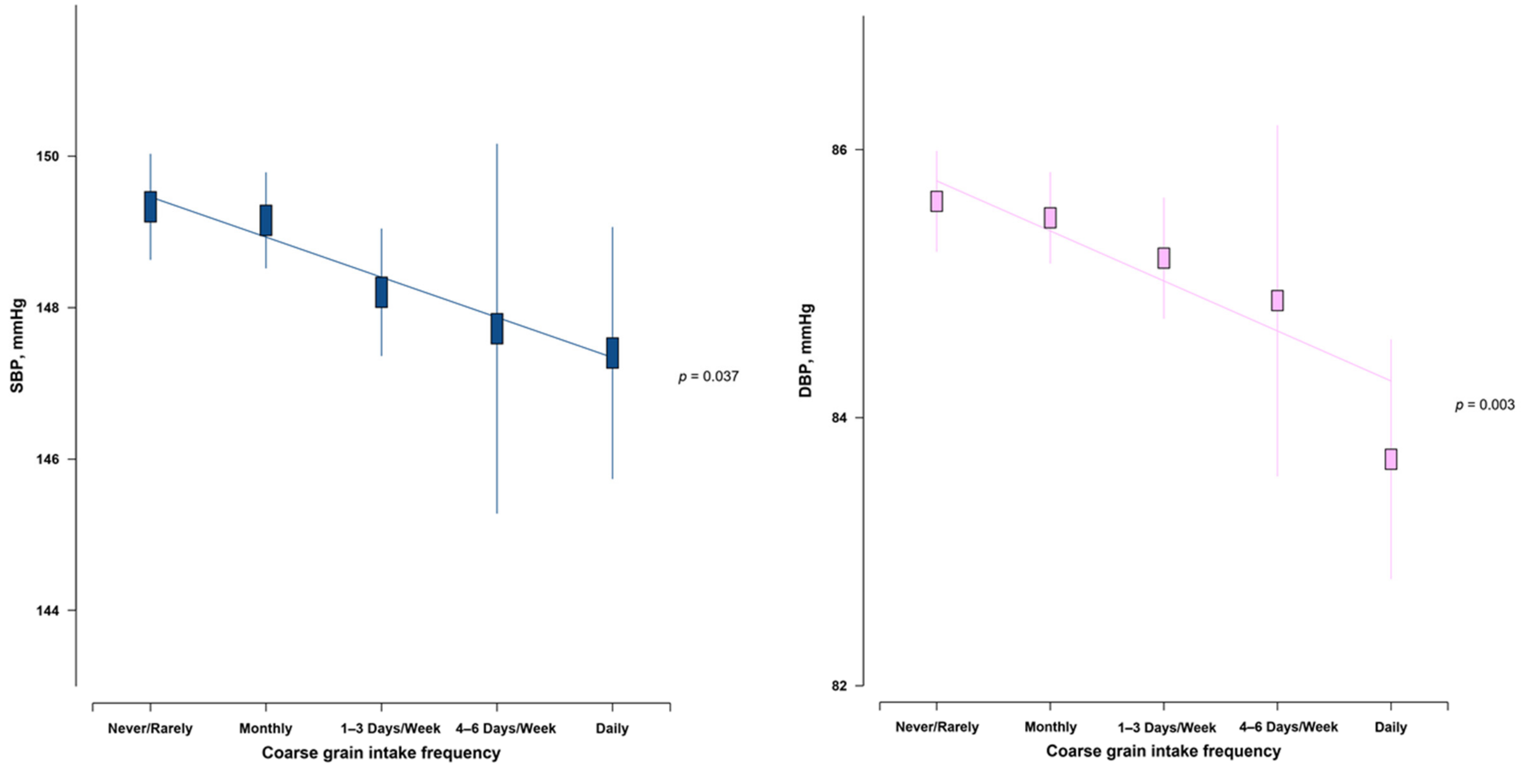

Supplemental Figure S3 Adjusted SBP and DBP by coarse grain intake frequency among diagnosed hypertension patients within 3 years before baseline (n=34163).Values were means adjusted for age, study area, education level, smoking, alcohol drinking, physical activity level, BMI, and local outdoor temperature.

Supplemental table S1 Adjusted odds ratios of undiagnosed hypertension by coarse grain intake frequency with additional exclusion criteria.

	Odds ratio (95%CI)					
	Never or rarely	Monthly	1–3 Days/Weeks	4–6 Days/Weeks	Daily	*p* for trend
Participants from Henan excluded (*n* =382,714)						
Model 1	1.00	0.97 (0.96–0.99)	0.92 (0.89–0.94)	0.86 (0.79-0.94)	0.76 (0.86–0.94)	<0.001
Model 2	1.00	0.97 (0.95–0.99)	0.91 (0.89–0.94)	0.85 (0.78–0.93)	0.75 (0.70–0.82)	<0.001
Participants with diabetes and cardiovascular disease excluded (*n*= 406,652)						
Model 1	1.00	0.98 (0.96–1.00)	0.92 (0.89–0.95)	0.89 (0.81–0.97)	0.78 (0.72–0.84)	<0.001
Model 2	1.00	0.97 (0.95–0.99)	0.91 (0.89–0.94)	0.86 (0.78–0.95)	0.79 (0.73–0.86)	< 0.001

Model 1, Adjusted for age, sex, and study area; Model 2, Additionally adjusted for education level (no formal education, primary school, middle/high school, or college/university), smoking (never, occasional, ex-regular, or regular), alcohol drinking (never, occasional, ex-regular, or regular), physical activity (MET-hr/day), BMI, and local outdoor temperature;.
